# A gloves-associated outbreak of imipenem-resistant *Acinetobacter baumannii* in an intensive care unit in Guangdong, China

**DOI:** 10.1186/s12879-015-0917-9

**Published:** 2015-04-11

**Authors:** Dan Ye, Jinglan Shan, Yongbo Huang, Jianchun Li, Changan Li, Xiaoqing Liu, Weiqun He, Yimin Li, Pu Mao

**Affiliations:** Department of Infection Control, The First Affiliated Hospital of Guangzhou medical university, Guangzhou, Guangdong 510120 China; State Key Laboratory of Respiratory Disease, The First Affiliated Hospital of Guangzhou medical university, Guangzhou, Guangdong 510120 China; Intensive Care Unit, The First Affiliated Hospital of Guangzhou medical university, Guangzhou, Guangdong 510120 China

**Keywords:** Outbreak, *Acinetobacter baumannii*, ICU, Gloves

## Abstract

**Background:**

Imipenem-resistant *Acinetobacter baumannii* (IRAB) is an important cause of hospital-acquired infection. We aimed to describe an outbreak of IRAB infection and to investigate its possible source in an intensive care unit.

**Methods:**

An environmental investigation was undertaken. Antimicrobial susceptibility testing was performed by microdilution. These isolates were genotyped by use of repetitive extragenic palindromic polymerase chain reaction (rep-PCR; DiversiLab). The study included 11 patients infected with IRAB and 14 control patients free of IRAB. Case and control patients were compared for possible predisposing factors. A multifaceted intervention was implemented to control the outbreak.

**Results:**

Thirty-nine IRABs were isolated from patients and the environmental surveillance culture in August, November, and December 2011. All isolates were resistant to imipenem. The IRAB strains belonged to seven clones (A–G) by the use of rep-PCR. There were four epidemic clones (D–G) in the outbreak, and Clone D was predominant. For the case–control study, patients with chronic obstructive pulmonary disease were susceptible to infection with IRAB. The hospital mortality of the case group was significantly higher than that of the control group.

**Conclusions:**

The outbreak strains were transmitted among infected patients and equipment by inappropriate use of gloves. A combination of aggressive infection control measures is essential for preventing recurrent nosocomial outbreaks of IRAB.

## Background

*Acinetobacter baumannii*, a non-fermenting Gram-negative bacterium, is recognized as an important opportunistic pathogen, and is particularly associated with mortality in intensive care units (ICUs) [1]. As a result of the simplicity of its growth requirements and its remarkable capacity for extended survival on environmental surfaces, *A. baumannii* is ubiquitous in the environment [2]. Thus, environmental contamination is an important source of cross-infection [3,4]. The carbapenem group of antimicrobial agents is commonly used for treating nosocomial infections caused by *A. baumannii* [5]*.* However, carbapenem resistance has been increasingly identified in the past decade [6,7], and imipenem-resistant *A. baumannii* (IRAB) has also been increasingly reported as a cause of nosocomial outbreaks [8-12].

On August 9, 2011, an outbreak of nosocomial infection with IRAB was noted in our medical ICU. In this study, we isolated IRAB from clinical specimens and the hospital environment, using the DiversiLab repetitive extragenic palindromic sequence-based PCR (rep-PCR) to assess the genetic relationship of these resistant isolates.

## Methods

The study was approved by the institutional Research Ethical Board of the First Affiliated Hospital of Guangzhou Medical University (2010082) for implementing outbreak control interventions and collecting clinical data retrospectively.

### Hospital settings

**Figure 1:**
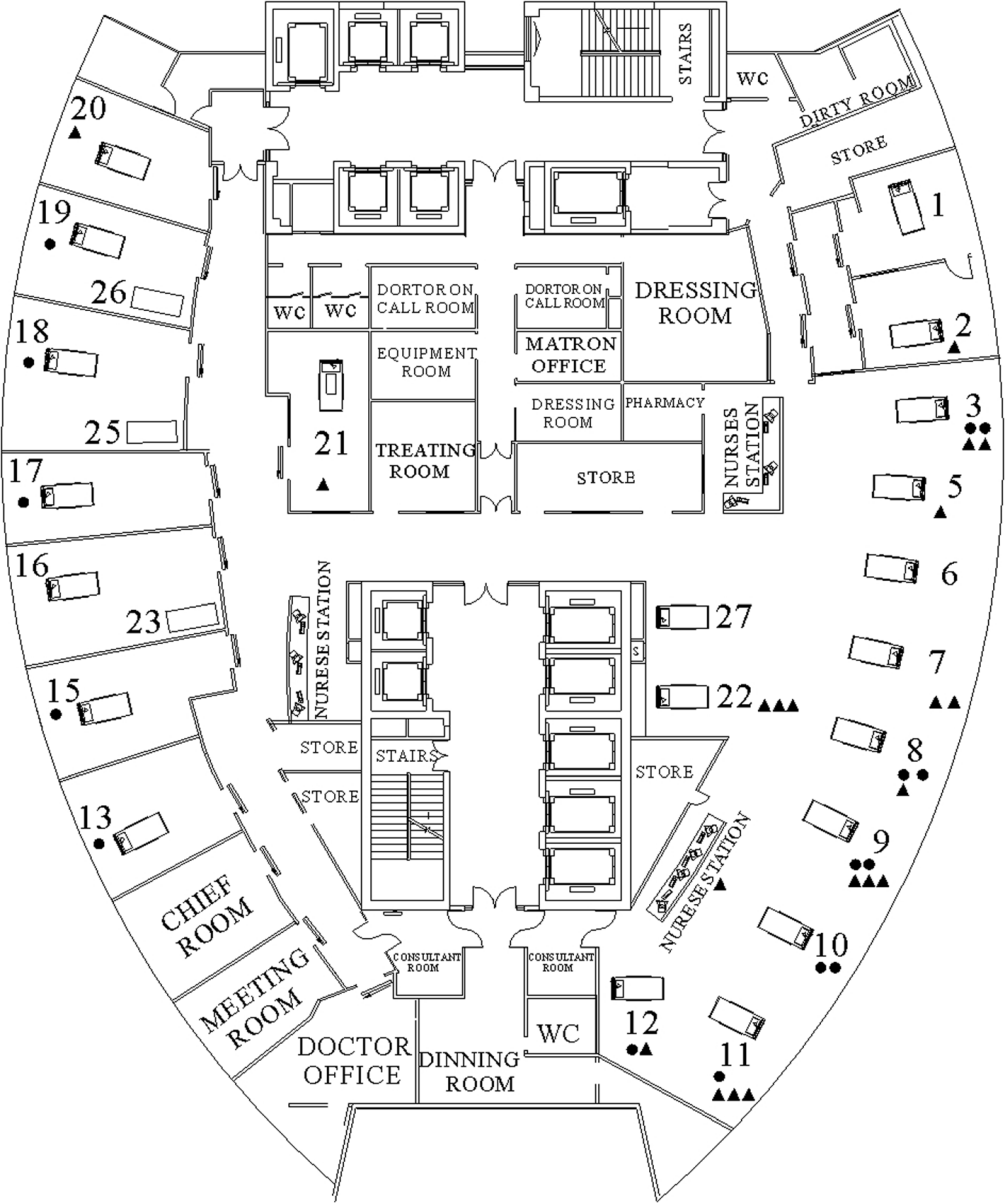
Schematic map of ICU showing the locations of samples that were positive for IRAB. (●) Patients with isolates positive for IRAB during environmental surveys in August, November, and December, 2011. (▲) Environmental samples positive for IRAB during environmental surveys in August, November, and December, 2011.

The First Affiliated Hospital of Guangzhou Medical University is a 2,000-bed, teaching hospital, and a tertiary referral center for major respiratory diseases in Southern China. In 2011, the annual number of admissions to the First Affiliated Hospital of Guangzhou Medical University was 45,020 and there were 560 admissions to the ICU. The outbreak occurred in a 27-bed medical ICU, where the nurse-to-patient ratio was 1–1.5:1 during daytime and 1:3 at night. A map of the ICU is shown in Figure 1.

### Epidemiological investigation

The outbreak period was defined as August 1–23. Nosocomial infection was defined according to the Centers for Disease Control and Prevention (CDC) [13]. For the case–control study, the cases were defined as those who had stayed in the ICU for at least 48 h with IRAB infection. The controls were patients without identification of IRAB after screening twice during the study period. Case and control patients were screened twice every week.

Demographic (age and sex) and clinical data (duration of ICU and hospital stay, sites of infection or colonization, underlying diseases, diagnosis, antibiotic classes used, mechanical ventilation, blood transfusion, hemodialysis, SOFA score, APACHE II urinary catheter, nasogastric tube, central venous catheter, peripheral venous catheter, arterial catheter, and clinical outcome) were extracted from the patient medical records.

### Microbiological methods

Research assistants swabbed potential fomites in the patient environment during outbreaks. Cotton-tipped swabs were moistened with sterile saline and surfaces vigorously scrubbed. Potential fomites included ventilator control panel and sensor, bed rails, supply cabinets, computer keyboards and mice, curtains, air vents, door handles, supply carts, and mattresses.

Transtracheal aspiration specimens were inoculated onto blood agar and MacConkey agar and incubated at 37°C for 48 h in an air incubator. Environmental swabs were incubated overnight in fastidious broth and then subcultured onto blood agar and MacConkey agar. Identification was conducted using the VITEK2 Compact 30 System (bioMerieux). The antimicrobial susceptibility was determined by microdilution in accordance with the Clinical and Laboratory Standards Institute guidelines.

### Molecular typing

Epidemiological typing of isolates was performed by rep-PCR fingerprinting (DiversiLab System) as previously described. Data were sent via an individual secured website (https://gzmedical.diversilab.com/) and analyzed with DiversiLab version 3.5, using the Pearson correlation coefficient and the unweighted pair group method. The isolates clustered ≥95% was considered related.

### Infection control interventions

Standard infection control measures were reinforced during the early outbreak (August 1–11). These included re-emphasizing hand hygiene; using alcohol foam between patient contacts following five moments of hand hygiene practices as recommended by the World Health Organization; and the practice of sterile techniques for all invasive procedures.

The crisis control staff decided to implement the enhanced control interventions, after five new cases were diagnosed during the early outbreak. Enhanced control interventions were implemented. (1) A cohort strategy was implemented. Colonized/infected patients were separated into different areas. The newly admitted patients were also separated and treated by a different team of healthcare workers (HCWs). (2) Inappropriate wearing of gloves in the ICU was strictly prohibited, and violators were financially penalized. One month merit pay should be kept out to pay for the reeducation. (3) The ICU was divided into two zones for daily cleaning, with separation of the clinical area and the HCW living area. The cleaning and decontamination criteria were formulated. The performance of regular environmental cleaning procedures in every shift was evaluated. (4) Unnecessary transfer of patients from other units or surrounding hospitals was stopped. Patients no longer requiring intensive care were immediately transferred to another unit with contact precautions. (5) Contact precautions were practiced for all patients upon admission. Continuation of contact precautions during ICU stay depended on the detection of IRAB in the first culture result. (6) Education crisis control staff reported progress of the outbreak and emphasized infection control lapses during the doctors’ and nurses’ shifts. The cleaning staffs were retrained.

Written informed consent for participation in the study was obtained from all participants.

### Statistical analysis

Continuous variables were described as mean and standard deviation, or median and interquartile range if they showed a skewed distribution. Categorical variables were described with absolute percentages. Continuous variables were analyzed by the Student’s *t*-test or the Mann–Whitney *U* test for non-parametric distributions. The categorical variables were compared by using the Pearson *χ*^2^ test.

## Results

### Outbreak description

**Figure 2:**
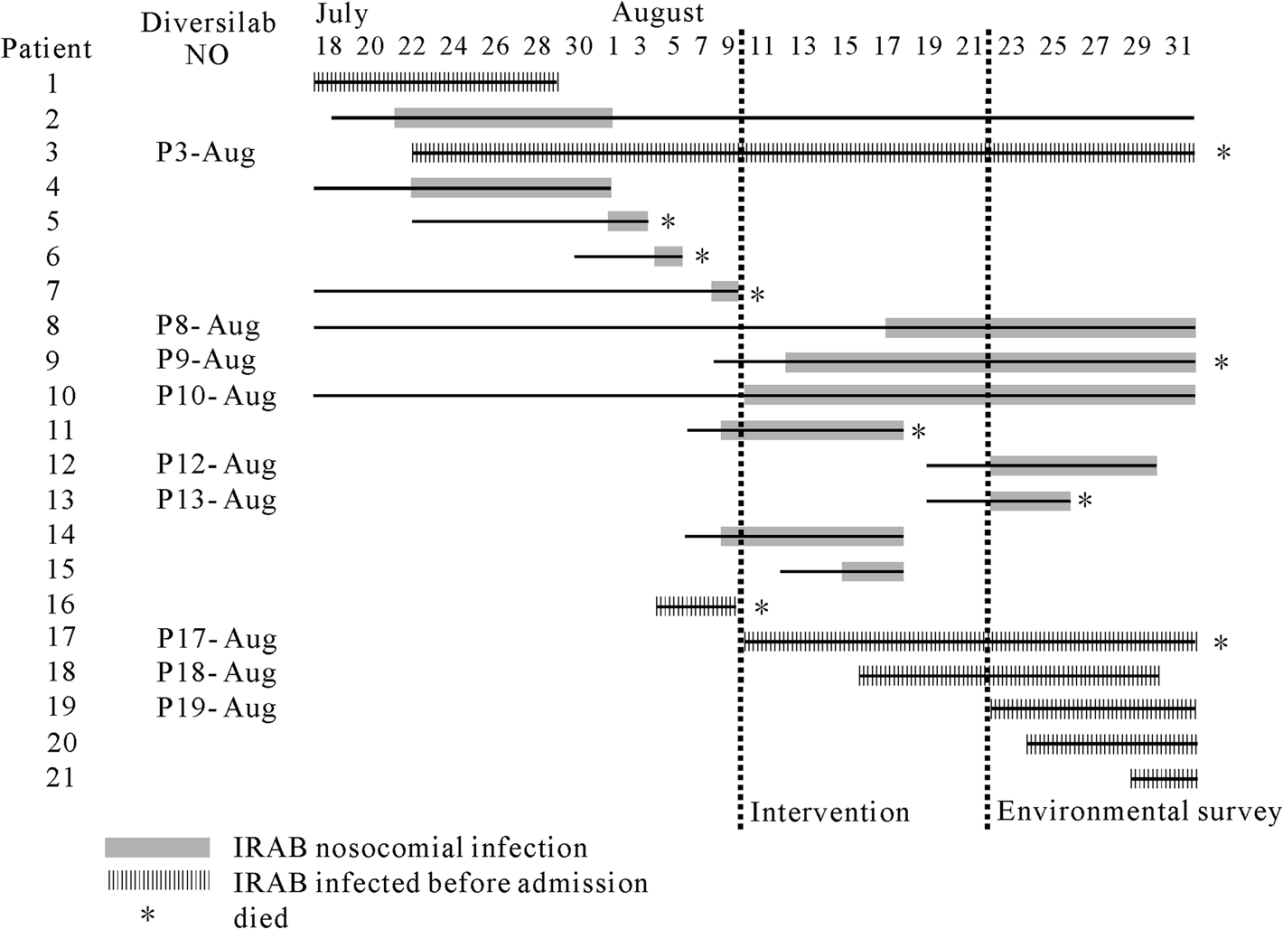
Progression of IRAB outbreak in the ICU. Line indicates the hospital stay periods in the ICU. The gray block indicates the period of IRAB nosocomail infection. The shaded block represents the patients who were infected with IRAB before ICU admission.

In mid-August 2011, the Infection Control Department was notified that there was an outbreak of IRAB infection. During the outbreak period, 11 patients had IRAB-positive cultures isolated from the lower respiratory tract. The mean age of affected patients was 65 years (range: 39–85 years). The antibiotic susceptibility patterns of the clinical isolates were identical, and the isolates were resistant to the majority of antibiotics, including carbapenems. An outbreak control team was established including ICU doctors and a head nurse, bacteriologists, infection control officer, and nurses. At the beginning of the outbreak, covert observation found that some doctors and nurses were wearing gloves all the time. The timeline of the outbreak investigation is illustrated in Figure 2.

### Isolation of IRAB

**Figure 3:**
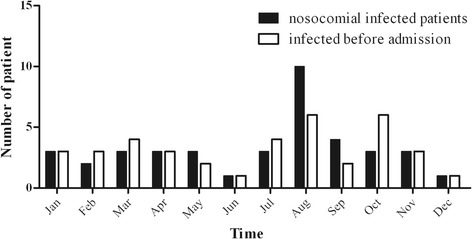
Monthly report of new cases of IRAB infection.

The distribution of patients with IRAB acquisition in the ICU and imported to the ICU during 2011 is shown in Figure 3. The number of patients that acquired IRAB in the ICU increased with the number of imported patients with IRAB. The majority of patients who were admitted to the ICU had pulmonary disease [severe pneumonia 16/25, and chronic obstructive pulmonary disease (COPD) 12/25], and all were treated with mechanical ventilation, therefore, we focused our analysis on bacteria isolated from transtracheal aspirates. These IRAB strains were also resistant to commonly used antibiotics, except polymyxin B and colistin. All of these strains were multi-drug resistant.

### Case–control study

The demographic risk factors for nosocomial acquisition of IRAB and outcome were compared between the case and control groups. The patients who were diagnosed with COPD upon admission to the ICU were predisposed to infection with IRAB (P = 0.028). The hospital mortality rate in the case group was higher than in the control group (P = 0.032) (Table 1).

**Table 1:** **Demographic and clinical characteristics of case patients infected with IRAB, compared with non-infected control patients**

**Characteristic** ^**a**^	**Cases (n = 11)**	**Controls (n = 14)**	**P value**
Age (years)	65.09 ± 12.65	63.86 ± 14.33	0.824
Gender, male (%)	5 (45.46)	5 (35.71)	0.622
Prior hospital stay (<1 year)	3 (27.27)	2 (14.29)	0.420
Length of stay in ICU (days)	6 (3–25)	11 (2.75–25.75)	0.784
APACHE II score upon ICU admission	21.82 ± 7.13	21.50 ± 8.04	0.919
SOFA score upon ICU admission	6.55 ± 3.14	7.00 ± 6.49	0.762
Primary ICU admission diagnosis			
Chronic pulmonary disease	8 (72.72)	4 (28.57)	0.028
Pneumonia	8 (72.72)	8 (57.14)	0.42
Diabetes mellitus	1 (9.09)	2 (14.28)	0.692
Hypertension	2 (18.18)	5 (35.71)	0.332
Malignancy	2 (18.18)	3 (21.42)	0.84
Surgery procedure (<30 days)	1 (9.09)	5 (35.71)	0.122
Antibiotic classes^b^			
Carbapenem	7 (63.63)	6 (42.86)	0.302
Penicillins	3 (27.27)	6 (42.56)	0.420
Piperacillin/tazobactam	5 (45.45)	6 (42.86)	0.897
Cefepime	7 (63.63)	6 (42.86)	0.302
Fluoroquinolones	3 (27.28)	3 (21.42)	0.734
Glycopeptide	6 (54.54)	8 (57.14)	0.716
Antifungal	4 (36.36)	4 (28.57)	0.678
ICU procedures			
Ventilator	11 (100)	14 (100)	--
Blood transfusion	5 (45.45)	7 (50)	0.821
Hemodialysis	0 (0)	4 (28.57)	0.053
Nasogastric tube	11 (100)	12 (85.71)	0.191
Venous central catheter	10 (81.82)	13 (92.86)	0.859
Peripheral venous catheter	11 (100)	13 (92.86)	0.366
Arterial catheter	4 (36.36)	6 (42.86)	0.742
Urinary catheter	11 (100)	14 (100)	--
Outcome			
28-day mortality	3 (27.27)	2 (14.29)	0.420
Hospital mortality	6 (54.56)	3 (21.42)	0.032

^a^All data are presented as the number, with the percentage in parenthesis, with the exception of age, APACHE II score, and SOFA score, which are presented as mean ± standard deviation. Length of ICU stay was presented as median (interquartile range).

^b^Case group: used within 30 days before the first IRAB isolate was discovered and the antibiotics had been used for at least 72 h. Control group: antibiotics had been used in the ICU for at least 72 h.

### Environmental and hand surveillance

In August 23, 310 environmental samples were collected. Nine (2.90%) samples were found to be culture positive for IRAB. These strains were isolated from computer keyboards (n = 3), bed rails (n = 3), nurses’ supply carts (n = 2), and a ventilator control panel (n = 1). The distribution of culture samples positive for IRAB from the environment and from patients is shown in Figure 3. Among 15 samples from the hands (or gloves) of 15 hospital care workers (doctors, nurses, and cleaners), four (26.67%) tested positive for IRAB; one of these was from the hands of a hospital care worker and three were from gloves. The samples from the HCW’s hands were collected without forewarning, and some were collected immediately after the HCW had been in direct contact with patients.

As a result of the continuing number of cases being reported, an environmental survey was conducted in November and December 2011. In November, 620 environmental samples were collected. At the same time, 10 (1.61%) samples were collected from the hands (or gloves) of hospital care workers. Twenty-one isolates were found to be culture positive for IRAB. These strains were isolated from bed rails (n = 3), computer keyboards (n = 2), curtains (n = 1), ventilator air flow sensor (n = 1), ventilator control panel (n = 1), supply cart (n = 1), and a bedside cabinet (n = 1). No samples from the hands (or gloves) tested positive for IRAB. In December, 620 environmental samples were collected, as well as samples from the hands (or gloves) of 10 hospital care workers, but only one isolate from a mattress was found to be culture positive for IRAB.

### Molecular typing of IRAB clinical and environmental isolates

**Figure 4:**
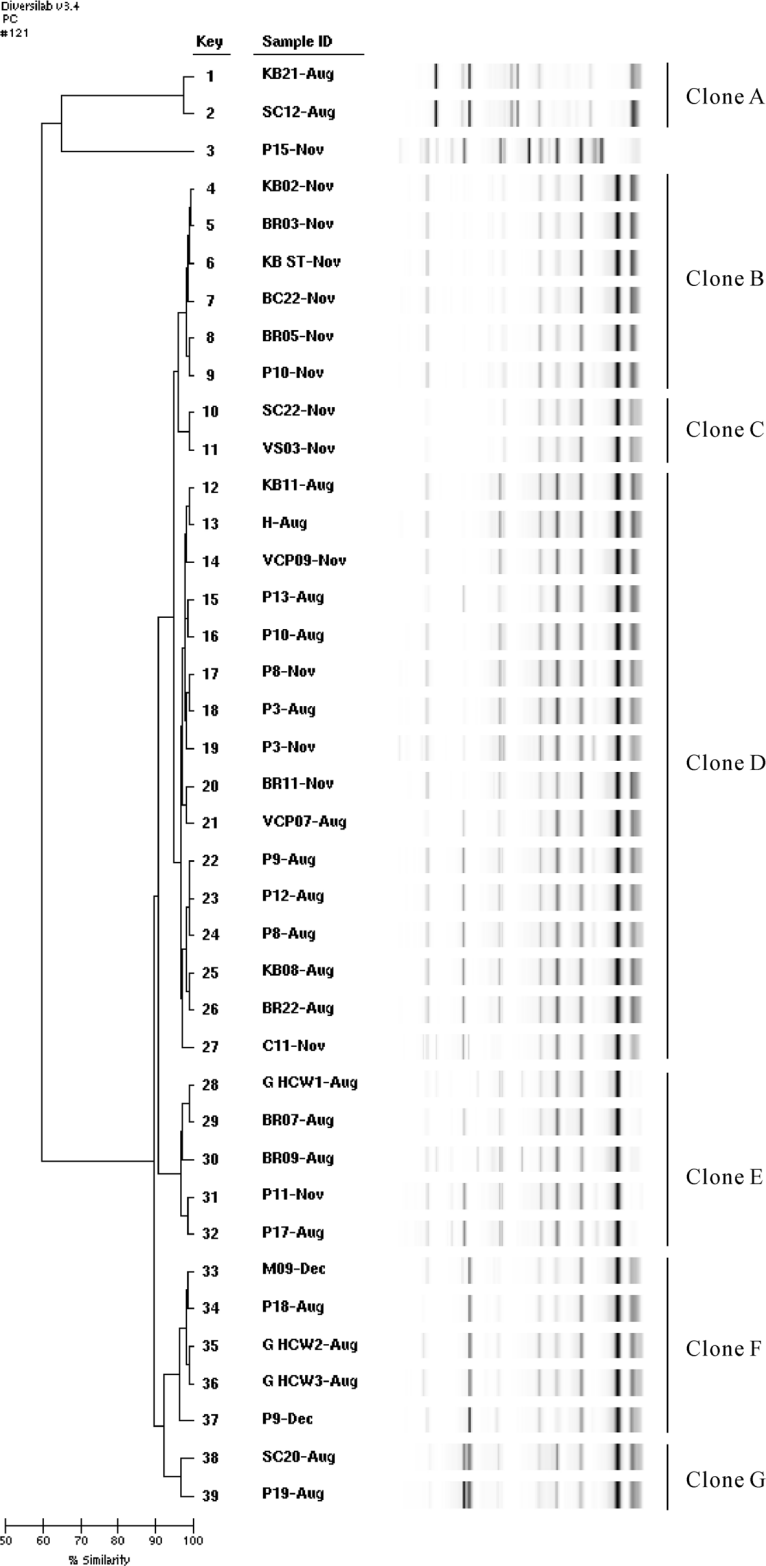
Rep-PCR-based similarity analysis and gel images for 39 IRAB isolates from clinical and environmental samples. Number presents the nember of beds. BC: bedside cabinet; BR: bed rail; C: curtain; G: gloves; H: hand; KB: computer keyboard; KB ST: computer keyboard at nurses’ station; M: mattress; P: patient; SC: supply cart; VCP: ventilator control panel; VS: ventilator air flow sensor.

We analyzed molecular typing of 39 strains, including 24 isolated from the environment in August, November, and December 2011; 15 of which were isolated from clinical samples during the environmental survey. DiversiLab revealed seven clones (designated A–G) (Figure 4). The most common was Clone D, which accounted for 16 (41.02%) of the 39 isolates. Clone D included eight clinical isolates, seven environmental strains, and one isolate from an HCW’s hand. These data suggest that Clone D was the predominant clone of the outbreak in the ICU.

## Discussion

Since 2008, there have been reports of ICU outbreaks caused by IRAB in our ICU [14]. The isolation rate of *A. baumannii* was 39.90% and the imipenem-resistance rate was 85%; IS*Aba1*-associated *bla*_OXA-23_ genes were prevalent in IRAB, with an isolation rate of 82.5% in 2011 (our unpublished data). Several IRAB nosocomial outbreaks with the IS*Aba1*-associated *bla*_OXA-23_ resistance mechanism have been reported [15]. Prevention of epidemic outbreaks of *A. baumannii* is a challenging task in our ICU.

It has been reported that computer keyboards and curtains are contaminated with bacterial pathogens, which can act as reservoirs of nosocomial pathogens [16-18]. Bed rails and supply carts are considered to be high-touch surfaces [19]. Furthermore, bed rails have been previously shown to have the highest frequency of contamination with *A. baumannii* [20,21]. In the present outbreak, environmental survey results revealed that bed rails were the objects most frequently found to be contaminated, followed by computer keyboards, and supply carts. Our findings were consistent with previously published data.

Covert observation found that some doctors and nurses wear gloves when contacting clean area items, such as operating the ventilator and computers. Therefore, we speculated that gloved HCWs contaminated equipment (such as computer keyboards, supply carts, and ventilator panels) with IRAB and disseminated the outbreak strains in the ICU. Swab cultures from the hands (or gloves) of HCWs found IRAB, and DiversiLab analysis showed that the strains isolated from the hands (or gloves), equipment, and clinical samples were Clones D–F. We identified transient hand (or glove) carriage, and confirmed that cross-transmission of IRAB was involved in hand contamination. We suggest that equipment was contaminated via the hands or contaminated gloves of HCWs. These data further suggest that the environment may be secondarily contaminated by patients, who then become a potential reservoir [22].

We impose financial penalties to prohibit contact the cleaning equipment with gloves. From the environmental culture results in December 2011, this intervention was efficient at preventing IRAB contamination, and the detection rate in environmental culture decreased from 2.90% (9/310) to 0.16% (1/620). This observation demonstrated that HCWs tend to wear gloves as a means of self-protection, rather than to prevent cross-infection of patients, which is consistent with hand hygiene using an alcohol-based hand rub [23].

We also analyzed homology of IRAB recovered from patients during the environmental survey. On August 23, 2011, nine infected patients stayed in the ICU. DiversiLab data demonstrated that six strains (P3, 9, 8, 10, 12, and 13 August) recovered from these patients belonged to Clone D. Five of these strains were isolated from nosocomially infected patients. One strain (P3-Aug) was recovered from a patient admitted on July 22, 2011, who had been infected with IRAB before admission, and cultured positively throughout admission. The remaining three clinical isolates (P17, 18, and 19 August) belonged to different clones that were isolated from patients infected with IRAB before admission and were transferred from another hospital. Therefore, we speculated that the original outbreak may have been from the patient with P3-Aug.

Epidemiological typing results suggested that the ICU had four epidemic clones (D–G) in the outbreak. The August clones remained in the environment and infected patients through to December 2011. It may be difficult to eradicate IRAB in ICU outbreaks [22], especially without closing ICU even with improved infection control practice [24,25]. Our intervention decreased the number of contaminated surfaces and resulted in a sustained reduction in the number of IRAB-infected patients.

Reduction the rate of nosocomial infection by surveillance and prevention program could be benefited from the Hawthorn effect [26,27]. Successful control outbreak depends on the cooperation of the ward staff [27]. In the present outbreak, covert observation had been executed which would influence the behavior of ICU staff. The outbreak control team including ICU doctors and nurse except bacteriologist and infection control officer. This can create a sense of ownership of infection control and then form cooperation among them.

Complete closure of clinical units has been recommended for outbreak control in some previous studies [28,29], but not in others [12,30,31]. In our setting, there was no available ICU to transfer the infected patients and we needed to keep the unit open. Therefore, our patients were immediately transferred out from the ICU once they were clinically stabilized, to reduce infection with IRAB and HCW workload.

Patients admitted to our ICU are often already infected (severe pneumonia was present in 16/25 patients). To control the outbreak, we implemented aggressive interventions of contact isolation for all patients after their admission, and the contact isolation was maintained until a negative culture result was obtained. By using enforced contact isolation, the chance of pathogens spreading during the time from patients’ admission to contact isolation can be reduced.

The present studies had some limitations. We demonstrated that the environment may be secondarily contaminated by the patients. To find the source of infection may need a long period of monitoring. The effects of HCW workload and the cost of extra cleaning were not investigated.

## Conclusion

The outbreak was controlled by a multidisciplinary approach. The study highlights the importance of glove removal. Contact isolation for all patients at admission may be an effective measure to avoid outbreaks of *A. baumannii* in endemic areas.

## Abbreviations

ICU: Intensive care units
IRAB: Imipenem-resistant *Acinetobacter baumannii*
rep-PCR: repetitive extragenic palindromic sequence-based PCR
HCWs: Healthcare workers
COPD: Chronic obstructive pulmonary disease
